# *Bacillus amyloliquefaciens* Probiotics Mix Supplementation in a Broiler Leaky Gut Model

**DOI:** 10.3390/microorganisms12020419

**Published:** 2024-02-19

**Authors:** Darwin Horyanto, Yadav S. Bajagai, Advait Kayal, Juhani von Hellens, Xiaojing Chen, Thi Thu Hao Van, Anita Radovanović, Dragana Stanley

**Affiliations:** 1 Institute for Future Farming Systems, Central Queensland University, Rockhampton, QLD 4702, Australiaadvait.kayal@cqumail.com (A.K.); 2 Bioproton Pty Ltd., Acacia Ridge, QLD 4110, Australiawendy@bioproton.com (X.C.); 3 School of Science, RMIT University, Bundoora, VIC 3083, Australia; 4 Faculty of Veterinary Medicine, University of Belgrade, 11000 Belgrade, Serbia; anita@vet.bg.ac.rs

**Keywords:** *Bacillus*, probiotics, gut microbiota, colonisation, broilers, dexamethasone

## Abstract

The supplementation of antimicrobial growth promoters (AGPs) has been banned in many countries because of the emergence of antimicrobial-resistant pathogens in poultry products and the environment. Probiotics have been broadly studied and demonstrated as a promising AGP substitute. Our study is centred on the effects of a multi-strain *Bacillus*-based probiotic product on broiler production performance and gut microbial profile in a dexamethasone-induced leaky gut challenge. Two hundred and fifty-six broiler chicks were hatched and randomly assigned into four groups (wheat-soybean meal basal diet (BD) = non-supplemented control (C), BD supplemented with dexamethasone in week 4 (CD), BD containing a probiotic from day one (P), and BD containing a probiotic from day one and supplemented with dexamethasone during challenge week 4 (PD)). The production performance and caecal, gizzard, jejunal lumen and jejunal mucosa swab microbiota were studied by 16S rRNA gene sequencing. The *Bacillus* probiotic product significantly improved production performance and altered caecal gut microbiota (*p* ≤ 0.05), but no significant impact on microbiota was observed in other gut sections.

## 1. Introduction

Global poultry production has increased significantly, particularly in Asia, accounting for 35% of global meat production [1]. The increase in per capita meat and poultry consumption has been the most substantial in countries that have experienced strong economic growth [2]. The expansion of intensive poultry production led to increased antimicrobial use, aimed at maintaining health and producing higher yields and better-quality products [3,4]. In 2017, 73% of global antimicrobials were used as growth promoters or as means of infection prevention in livestock, contributing to antimicrobial resistance (AMR) in pathogenic microorganisms [5,6].

The emergence of AMR implies that bacteria, currently sensitive to antibiotics, will become increasingly resistant, and infections will be harder to cure, leading to higher morbidity and mortality levels [7]. Wright [8] pointed out the indirect impact, stating that 90% of antibiotics applied in livestock are expelled via excreta and widely spread in the environment, such as soil and water. This dispersion significantly impacts the environmental microbiome and harms sustainability and human health [9].

Antimicrobial growth promoters have been banned in Europe since 2006, but several major poultry-producing countries still permit the use of AGPs, although they are being phased out due to consumer demands for antibiotic-free products [10]. Global antibiotic use was projected to increase by 8% by 2030, with the majority of intensity hotspots being in Asia [5]. The overuse or misuse of antibiotics and the lack of new antibiotic drug development pose a severe risk for human and livestock health [11]. Poultry products can also contain residual antibiotics [12]. Multiple authors observed high levels of AMR genes in the gut microbiota of both humans and livestock, including swine and poultry [8,13,14,15], and current molecular detection methods indicate the direct and indirect spread of AMR genes in livestock meat products consumed by humans [16].

Poultry is continually exposed to various physiological and pathological stressors in every stage of the production cycle [17,18,19]. This puts pressure on the poultry industry, particularly in the intensive broiler production sector, to achieve optimal performance and minimal economic losses, as well as ensure broiler meat safety via the elimination and control of zoonotic pathogens like *Salmonella* and *Campylobacter* in broiler birds [20].

A probiotic, by definition, is “a live microbial feed supplement which beneficially affects the host animal by improving its intestinal microbial balance” [21]. Probiotics that promote digestion, balance gut microbiota and maintain a healthy gastrointestinal (GI) environment [22] are potential substitutes for AGP [23,24]. Probiotics improve performance [24,25,26], digestibility [27,28], gut health and microbiota [29,30,31], pathogen inhibition [32,33], immunomodulation [34,35] and the gut mucosal immune system [36,37]. Despite the benefits, achieving precise application of probiotics in broilers is challenging. This shortfall stems from our lack of knowledge of the complex dynamics of the poultry gut microbiome and GI ecosystem [38]. For example, probiotic efficacy is highly dependent on taxonomy (genus, species and strain of bacteria), viability [39], stability post-pelleting and in poultry GIT, the application method, dosage level, diet, age of birds, biosecurity and environmental stressors [22]. On the other hand, some studies reported a minimal impact of probiotics on broiler performance [40] or that they improve body weight (BW) but have no impact on the feed conversion ratio (FCR) [41]; thus, the benefits of probiotics do not always manifest as performance advances.

In a study by Tran et al., (2023), multiple *Bacillus* strains were compared for their antimicrobial effects against common poultry pathogens, including *Clostridium perfringens*, *Escherichia coli*, *Pseudomonas aeruginosa*, *Staphylococcus aureus* and *Salmonella enterica*. The three *B. amyloliquefaciens* strains were selected for their stability and performance and combined into a multi-strain probiotic product used in this study [33]. Here, we present the study of the benefits of early colonisation of *Bacillus*-based probiotics on broiler production performance and GI microbiota challenged by the dexamethasone (DEX) leaky gut model.

## 2. Materials and Methods

### 2.1. Animal Ethics Statement

Central Queensland University’s Animal Ethics Committee approved the research presented in the document under approval 0000023123. All animal manipulations were performed according to the Australian Code for the Care and Use of Animals for Scientific Purposes and reported according to the guidelines and regulations of Animal Research: Reporting of In Vivo Experiments (ARRIVE).

### 2.2. Animal Trial

Assuming 80% hatchability, 320 broiler eggs (ROSS 308) were purchased from a commercial breeder (Woodlands, QLD, Australia). These eggs were incubated at 37.5 °C and 55–65% relative humidity (RH), and hatched at 21 days of age, producing 256 birds for the main experiment; late hatchlings and birds with any detected health issues were grown separately and not included in the study. Two hundred and fifty-six Ross 308 broiler chicks hatched in-house were randomly assigned into one of four experimental groups in a completely randomised 2 × 2 design for five weeks. Each experimental group had eight replicate pens (120 cm × 120 cm × 80 cm) of eight birds per pen (64 birds per group) with wood shavings as bedding material. The temperature, RH, and lighting program were maintained as per Ross 308 guidelines. Temperature and RH were set at 32 °C and 40% on days 1–7 and decreased by 1 °C weekly. A 23 L:1 D lighting program at 30–40 lux was applied on days 1–7 and continued by a 16 L:8 D lighting program at 10 lux.

The chicks were provided with a commercial BD (Red Hen Chick, Laucke Mills, Daveyston, South Australia) and, depending on probiotic and DEX supplementation, were labelled as follows: BD only = non-supplemented control (C), a BD supplemented with DEX during week 4 (CD), a BD containing probiotic from day one (P), and BD containing probiotic from day one and supplemented with DEX during challenge week 4 (PD). The BD was commercial chicken crumble (Red Hen Chick by Laucke Mills). Feed was nutritionally balanced for essential nutrients such as protein (23%), fat (5%), fibre (6.90%), energy, vitamins and minerals and had no antibiotics or coccidiostat. The probiotics were mixed every week and stored in a cool place. Throughout the study, birds had ad libitum access to feed and water.

The supplemented probiotic (Natupro NG, Acacia Ridge, Brisbane, Australia) was a mixture of three *Bacillus amyloliquefaciens* strains. The experimental diets P and PD contained the probiotic product supplemented at 500 g/t. This provides a desirable probiotic concentration of 3 × 10^8^ colony-forming unit per kg (CFU/kg) in feed.

### 2.3. DEX Challenge Period

Birds in CD and PD groups were supplemented with DEX at a concentration of 0.6 ppm from days 28–35, inducing gut barrier dysfunction following the protocol optimised by Vicuña et al. [42]. Experimental design in the DEX challenge week from day 28 to day 35 was 2 × 2 factorial. The stock solution of DEX was prepared daily by dissolving DEX (Sigma-Aldrich, St. Louis, MO, USA) in 70% ethanol, achieving a concentration of 0.6 ppm DEX per kg of feed. The preparation involved spraying DEX solution onto the feed while mixing in a mixer. Throughout the one-week DEX supplementation period, various parameters were closely monitored and collected daily, including BW, body weight gain (BWG), feed intake (FI), FCR, mortality, and overall health status of broiler birds (e.g., diarrhoea). The scoring for assessment of intestinal pathology was used every day of the DEX challenge, and the parameters scored included inactivity, hunched posture, ruffled feathers, rate of breathing, crusty eyes, shivering, diarrhoea, rectal bleeding, not inquisitive or alert and weight loss. The presence of abnormal behaviour was also recorded, as per the scoring sheet approved by the Animal Ethics Committee. Litter samples were collected from each pen at the end of the challenge for moisture content measurements.

### 2.4. Broiler Performance

From days 0 to 28, performance parameters, including BW, BWG, FI, FCR and mortality, were collected weekly. From days 28 to 35, during the DEX challenge, performance parameters were collected daily. Overall production performance was calculated for the whole study period.

### 2.5. Sample Collection

On day 35, three broilers per pen (24 birds per group) were euthanised by CO_2_ gas. The weight was collected with 0.1 g precision, the carcass was opened, and GIT content was sampled from the gizzard, jejunum, and caecum, then stored at −80 °C. Jejunal mucosal swabs were collected from the mid-section between the posterior end of the duodenal loop and Meckel’s diverticulum. Liver weight was also collected. Feed samples for microbiota analysis for each experimental group were collected weekly post-mixing process and mixed well. All samples were stored at −80 °C before being processed for DNA extraction.

### 2.6. DNA Extraction, Amplification and Sequencing

DNA from the gizzard content, jejunum content, caecum content, and jejunal mucosal swabs (*n* = 96 each) and feed samples were extracted using the DNA mini spin column (Enzymax LLC., CAT# EZC101, Lexington, KY, USA) following the lysis protocol for digesta and fecal samples suggested by Yu and Morrison [43]. DNA concentration was measured using a Nanodrop™ One Spectrophotometer (ThermoFisher Scientific, Wilmington, DE, USA). The sequencing library was prepared by amplifying the V3-V4 region of the 16S rRNA gene using primer pairs pro341F (5′-CCTACGGGNBGCASCAG-3′) and pro805R (5′-GACTACNVGGGTATCTAATCC-3′) with index, heterogeneity spacer and Illumina sequencing linkers [44]. The library was purified using AMPure XP Kits (Beckman Coulter, Brea, CA, USA) and sequenced with the Illumina Miseq platform with paired-end configuration (2 × 250 bp).

### 2.7. Bioinformatics

Cutadapt [45] was used to demultiplex the raw sequences and the better quality reads with a minimum Phred score of 25 across the length of 200 nt were analysed with Quantitative Insights into Microbial Ecology 2 (QIIME2) [46]. Quality filtering, denoising, and chimera removal were performed using Dada2 [47] plugin and the taxonomy was assigned with the SILVA v 138.1 database [48].

Further downstream statistical analysis and visualisation were carried out using a range of packages, including Phyloseq [49], Phylosmith [50], Vegan [51], and Microeco [52] in R.

### 2.8. Histology

For histomorphological analysis tissue, samples from the ileum were collected and fixed in 10% neutral buffered formalin solution. All processing (paraffin embedding, 4 μm thickness slide sectioning and staining) was performed in the Veterinary Laboratory Services at The University of Queensland, Gatton, Australia. Slides were stained using the Periodic Acid–Schiff–Alcian Blue staining method, and scanned using Panoptiq™ software (ViewsIQ Inc., Vancouver, BC, Canada) and a Nikon Eclipse Ci-L Plus biological microscope (Nikon Corporation, Minato-ku, Japan). Villus height, villus width, crypt depth, and the number of goblet cells were measured from 6 randomly selected well-positioned villi per slide and six slides per group. Morphometric analysis of the ileum was performed using Olympus software SensEntry v1.13.

### 2.9. Performance Analysis

The broiler performance, including BW, ADG, ADFI, FCR, was analysed by one-way ANOVA by GraphPad Prism (10.0). The normality and homogeneity of variance were confirmed before applying statistical analysis. Multiple (pair-wise) comparisons of means were analysed by post-hoc Tukey’s HSD.

## 3. Results

### 3.1. Animal Performance

Broiler performance is presented in Table 1. Broiler BW showed no significant variation among the groups (*p* > 0.05), except on day 21, where probiotic groups had 25.11 g (2.84%) more BW (*p* < 0.05) versus the control groups. A similar performance pattern is seen in ADG and FI on days 14–21. There is 3.06 g/day (4.57%) improvement in ADG (*p* < 0.05) and 4.09 g/day (4.90%) in ADFI (*p* < 0.001) of the probiotic groups. No significant difference is evident in days 0–7, 7–14, 21–28 and 0–28. There were no significant differences in FCR (*p* > 0.05), except on days 0–28, when the probiotic groups had a 0.02 point (1.56%) improvement in FCR (*p* < 0.05). The probiotic groups had lower mortality (1.56% vs. 3.13% in control groups).

**Figure 1:**
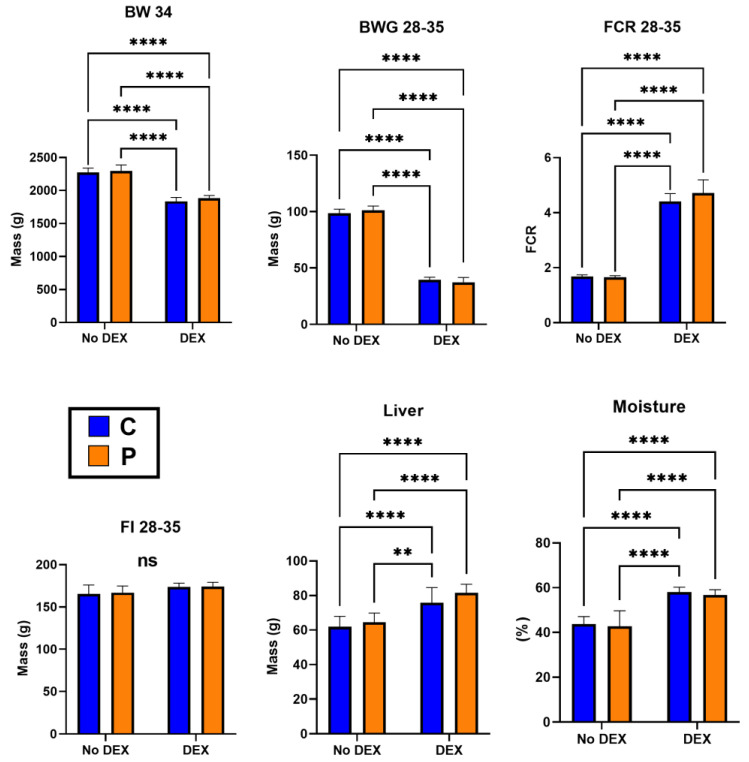
Broiler performance, liver weight and litter moisture parameters post-DEX period (days 28–35); **** *p* ≤ 0.0001 and ** *p* ≤ 0.01; ns = no significant comparisons.

The scoring of birds for intestinal pathology observations showed no significant difference in the following parameters: inactivity, hunched posture, ruffled feathers, rate of breathing, crusty eyes, shivering, rectal bleeding, not inquisitive or alert and weight loss. However, the diarrhoea scores were significantly higher in DEX birds compared to unchallenged birds (*p <* 0.001). This was confirmed with a significant difference in litter moisture levels (DEX vs. NoDEX *p* < 0.0001, Figure 1), in agreement with the original leaky gut model validation by Vicuña et al. [42].

Table 2 and Figure 1 present broiler performance on days 28–35 (DEX period). There is a significant difference (*p* < 0.05) between the DEX-challenged groups (CD and PD). Broilers’ BW, BWG and FCR decreased by DEX, but the probiotic-supplemented groups (C vs. P and CD vs. PD) had no significant improvement (*p* < 0.05). The BW of probiotic-supplemented birds was improved by 0.93% in non-DEX and 2.57% in DEX groups. The FI was similar across experimental groups (*p* > 0.05), and mortality was constant in C vs. P (0%) and CD vs. PD (1%) groups. Higher liver weights were observed in DEX groups (*p* < 0.05) (Figure 1).

### 3.2. Overall Structure of Microbial Communities

**Figure 2:**
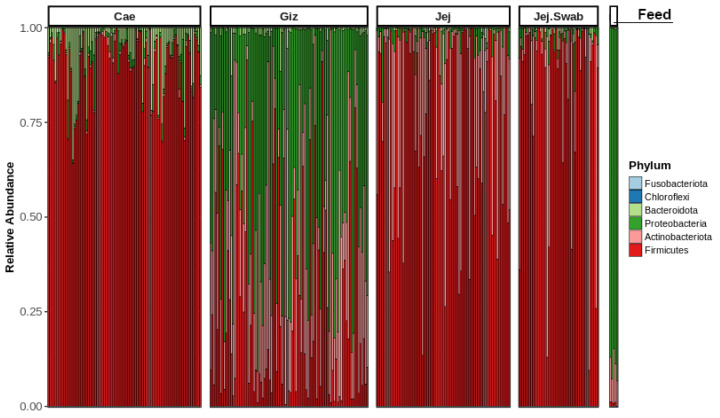
Phylum-level taxa distribution across sampled intestinal origins.

Figure 2 shows the distribution of phyla among various sample origins, including the gizzard, jejunum, jejunal mucosa, caecum, and feed. The most abundant phyla observed were *Bacteroidota*, *Proteobacteria*, *Actinobacteriota* and *Firmicutes*. The upper GIT segment (gizzard) was populated by *Proteobacteria*, the small intestine (jejunum) was dominated by *Actinobacteriota* and *Firmicutes*, and the large intestine (caecum) was dominated by *Firmicutes*. The abundance of *Proteobacteria* in the gizzard may have originated from the feed microbiota, where *Proteobacteria* dominate.

**Figure 3:**
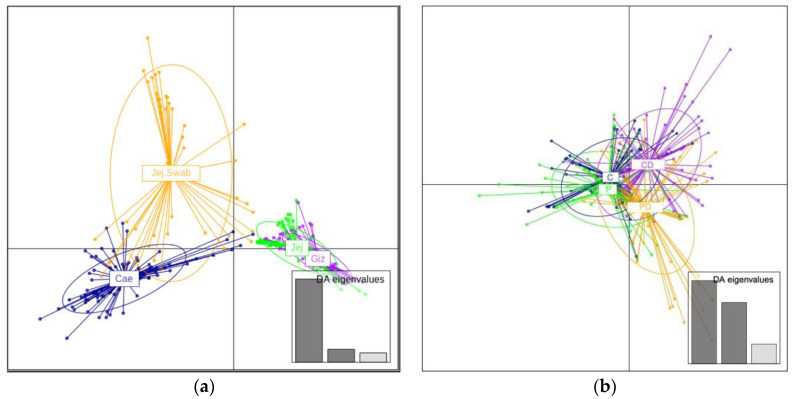
The genus-level DAPC plots are presented as follows: (**a**) the left panel displays a genus-level DAPC plot of samples from different origins, each depicted in different colors; (**b**) the right panel illustrates a genus-level DAPC plot of different experimental groups in broilers, each depicted in different colors.

The DAPC (Discriminate Analysis of Principal Components) plot of genus-level data in Figure 3 clearly separates different sample origins (Figure 3, left panel). The DAPC plot of different experimental groups shows similarities in unchallenged C and P. However, the DEX-challenged probiotic-supplemented and unsupplemented groups were separated (CD and PD, Figure 3, right panel), indicating the difference in community response to leaky gut challenge.

#### 3.2.1. Alpha Diversity

**Figure 4:**
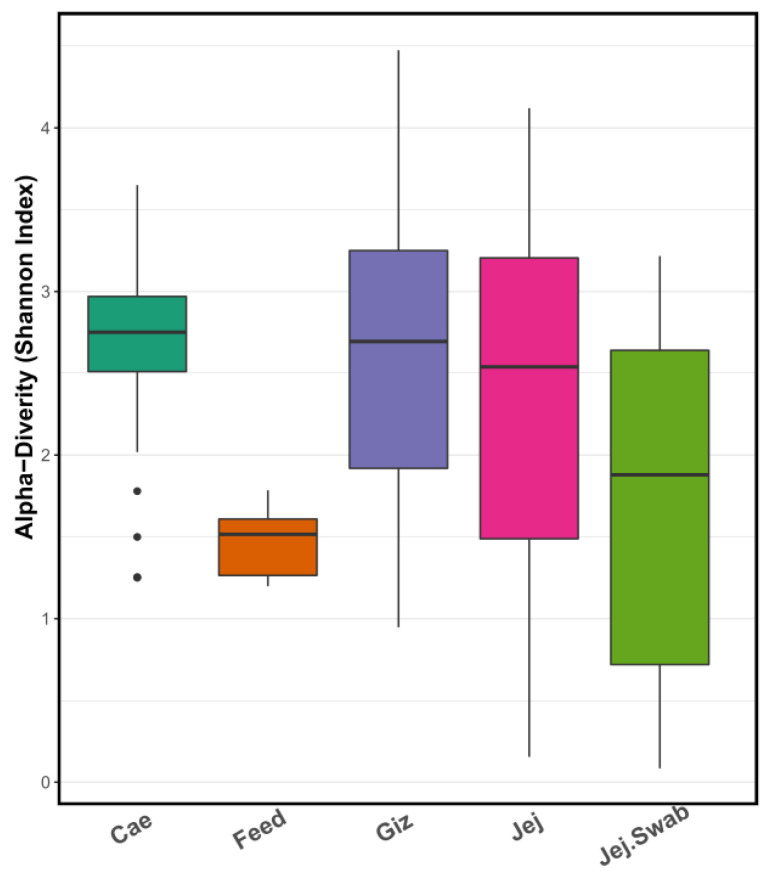
Alpha diversity (Shannon index) plot demonstrates high caecal microbiota diversity, moderate gizzard, jejunal and jejunal swab microbiota diversity, and lower feed microbiota diversity, each depicted in different colors.

As expected, alpha diversity, presented as the Shannon index, was different between gut sample origins (Figure 4). The gut samples from the same groups were individually analysed and averaged for each sample origin. The caecum showed the highest diversity, whereas the gizzard, jejunal content and jejunal mucosa swab samples presented similar diversity. Feed samples in our study showed the lowest alpha diversity.

#### 3.2.2. Beta Diversity

**Figure 5:**
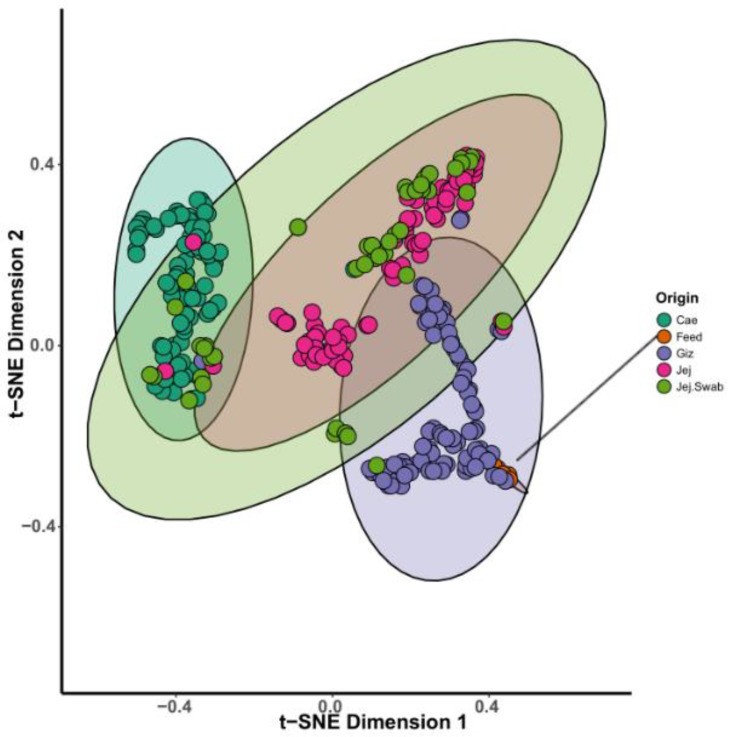
The t-SNE plot demonstrates the grouping of sample microbial profiles based on individual sample origin.

The beta diversity analysis, complemented by multivariate models, shows sample-to-sample similarity and potential grouping based on the origin and experimental group. Notably, non-metric multidimensional scaling (NMDS), principal coordinate analysis (PCoA), and stochastic neighbour embedding (t-SNE) plots pointed out a clear separation by origin, as demonstrated in t-SNE, a statistical method adept at visualising high-dimensional data. Figure 5 visually depicts the distinctiveness of separation and close grouping observed in the bird’s GIT. For example, caecal and gizzard samples created distinct clusters, and as expected, strong overlap is evident in the jejunal content and jejunal swab samples. Feed samples were embedded in the gizzard cluster, which suggests a significant influence of feed microbiota on the overall microbiota detected in the gizzard. Therefore, gizzard microbial community analysis should focus on identifying and understanding the specific differences in the microbiota instead of commenting on overall bacterial membership (Figure 5).

### 3.3. Gut Section-Specific Response

#### 3.3.1. Caecum

None of the C, P, CD and PD groups exhibited significant variations in caecum alpha diversity. However, in beta diversity analysis, PERMANOVA of both weighted and unweighted Unifrac showed significant differences among the DEX and non-DEX groups (Table 3). Moreover, paired MANOVA (Table 4) in the caecum demonstrated overlap between C and P groups and CD and PD groups, indicating that DEX had a different impact on beta diversity in broilers based on both weighted and unweighted Unifrac metrics.

**Figure 6:**
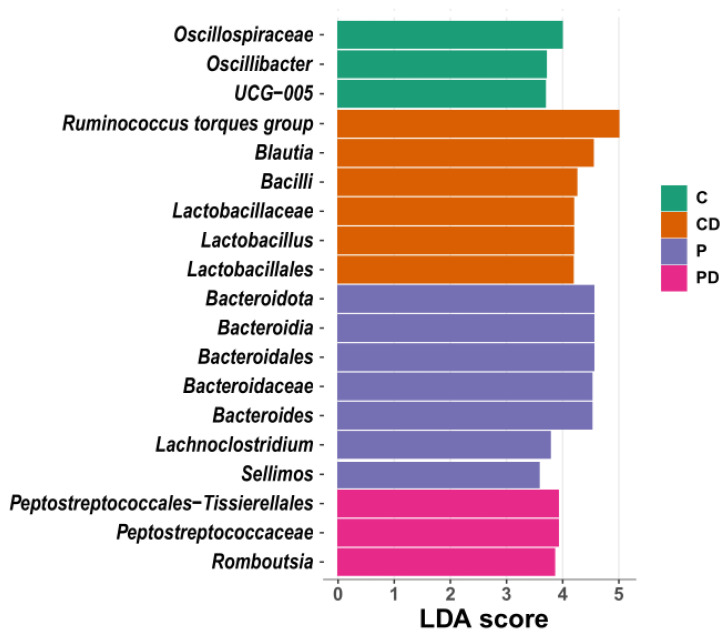
LEfSe presenting taxa at all taxonomic levels in caecum microbiota (*p* < 0.05 and LDA > 3.5). LDA = linear discriminatory analysis; LEfSe = LDA effect size.

The differential taxa in each gut origin analysed with the linear discriminant analysis effect size (LEfSe) biomarker discovery tool are shown in Figure 6. The caecum was characterised by the abundance of *Oscillospiraceae*, *Oscillibacter* and *Ruminococcaceae UCG-005* in C; *Blautia*, *Bacilli* and *Lactobacillus* in CD; *Lachnoclostridium* and *Bacteroides* in P; and *Romboutsia* and *Peptostreptococcaceae* in the PD group (Figure 6).

While there were no significant differences between probiotic-supplemented and non-supplemented groups (C vs. P), there were significant differences between CD and PD by both weighted and unweighted Unifrac (Table 4), indicating that probiotic supplementation significantly altered cecal microbiota response to the leaky gut challenge despite of not showing any significant effects under ideal growth conditions.

#### 3.3.2. Gizzard, Jejunum and Jejunal Swab

No major differences among the experimental groups were observed in alpha diversity matrices in samples collected from the gizzard, jejunum content or jejunal mucosa swabs. To assess the beta diversity as a community structure measure, two-way PERMANOVA was performed, and paired MANOVA assessed individual comparisons; however, there were no significant differences in any of the comparisons in the gizzard, jejunum and jejunal mucosa microbiota.

### 3.4. Histological Analysis

**Figure 7:**
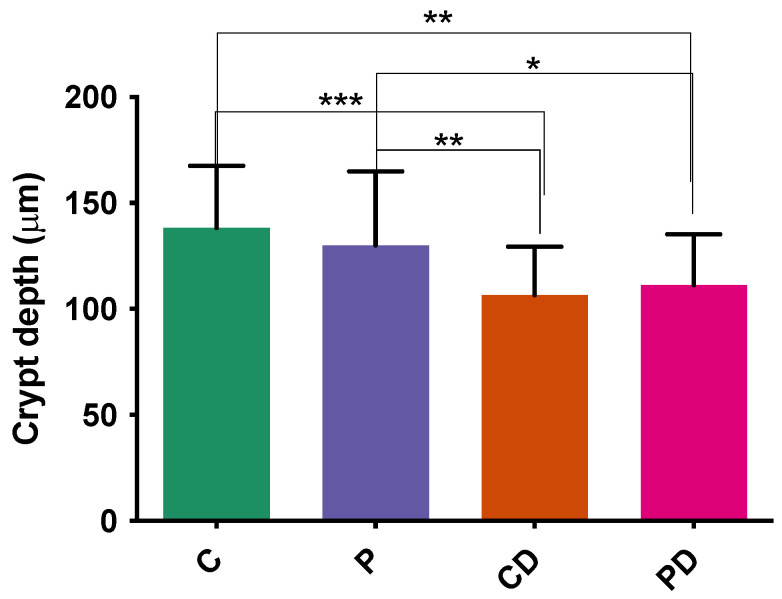
Crypt depth (μm) of different experimental groups in ileum. *, **, *** Statistically significant *p* < 0.05.

The histomorphological analysis revealed that the ileum exhibited no significant differences in villus height and villus area across all experimental groups. The crypt depth was significantly lower without a sign of degeneration in both DEX groups compared to no DEX groups (Figure 7). The number of goblet cells did not differ among the groups.

## 4. Discussion

Antibiotics and genetic selection practices have modified microbial diversity, including specialised microbiota and potential health and metabolic benefits in broilers [53]. The success of a probiotic in promoting broiler performance stems from a balanced and complex gut ecosystem, resulting in improved digestion, GIT environment and overall broiler health. Therefore, our primary objectives were to analyse gut microbiota composition and its importance in broilers and assess growth performance parameters. Generally, daily supplementation of 10^8^–10^9^ CFU has been a successful common practice for most probiotics [54]. In a previous study by Mountzouris et al. [55], supplementing a five-bacterial strain probiotic product at 10^9^ CFU/kg improved broiler performance and FCR, as well as modulating caecal microbiota composition and metabolic activities (e.g., VFA, protein concentration and microbial glycolytic enzyme activity) in broilers. In our study, broiler performance parameters, including BW, ADG, ADFI and FCR, were significantly improved in the probiotic-supplemented birds demonstrating growth-promoting effects.

*Bacillus* produce glycolytic enzymes, which convert glucose-6-phosphate and NAD^+^, producing more ATP [55]. Our study agrees with the similar growth-promoting impact of probiotics reported in broilers by various studies [25,33,34,56,57]. However, there were also studies where positive effects on performance were not recorded [40,58]. Reliable comparison between the studies is challenging because of large protocol variations influencing probiotic application and experimental design [54]. These variations include composition and viability of probiotic species, level of dosage applied, method of application (e.g., once, continuous, spraying, live inoculum), age of birds, hygiene conditions, and environmental stressors (e.g., heat stress, diseases) [55].

The present study confirmed that the DEX application had induced significant challenges in broilers. This is confirmed by severe diarrhoea and poor performance indicators, such as BW, BWG and FCR, in DEX groups (CD and PD), with comparable mortality. This was anticipated and consistent with previous studies [42,59]. As an exogenous glucocorticoid, dexamethasone significantly impacts glucose metabolism and energy homeostasis in various organs [60], impairing broilers’ performance, as observed in the current study. DEX also exhibits preferential binding in Type II GC Receptors, inducing physiological stress like immunosuppression [61]. This profound impact of stress-induced immunosuppression by DEX on immune organs is evident via the enlarged liver, as seen in our study where the CD and PD groups had 14–17 g heavier livers, similar to those previously reported by Barekatain et al. [62]. DEX was reported to modify liver morphology and alanine aminotransferase (ALT) concentration in broilers, indicating liver damage [61]. Furthermore, villus height, villus area, and goblet cells of ileum showed no statistical difference across all experimental groups, except that crypt depth was surprisingly decreased in the DEX groups. The crypt depth is a standard indicator of gut health in poultry. The crypt is known as a villus producer, and a bigger crypt indicates quicker tissue turnover [63]. The decreased crypt depth in our study was evident in both DEX groups; shallower crypts can be a sign of impaired cell proliferation or damage to the tissue that agrees with the DEX challenge [64]. Different outcomes of gut histomorphological analysis after probiotic supplementation were also seen in various studies [65].

Both positive [66] and minimal improvement [67] of the gut barrier by *Bacillus* probiotics supplementation have been documented. The potential beneficial impact of probiotics and changes in gut microbiota is believed to be linked, but in our study, minimal probiotic impact on days 28–35 during the DEX challenge period could be explained by housing conditions, composition of the BD, and viability of probiotic organisms. Strain-specific and disease-specific impacts of probiotics on gut barrier integrity [68] may contribute to minor observed impacts during the DEX challenge in our study. For example, in a similar study, Konieczka et al. [69], supplementing one-strain *B*. *licheniformis* and multi-strain *Bacillus*-based probiotics, observed marginal improvement in broiler performance in challenge conditions.

The colonisation in poultry is rapid, and gut microbiota maturation starts from the first days of life [70]. Probiotic colonisation is challenging as poultry GIT is short, and feed transit is generally between 3 and 4 h [71]. Thus, early colonisation with beneficial bacteria is crucial, particularly in broilers where the production cycle is rapid. As an AGP substitute, a good probiotic should encourage balanced gut microbiota conditions, promote the growth of other beneficial microorganisms, suppress harmful microorganisms, and improve gut health [55]. Our study produced a snapshot of microbiota distribution and sample-to-sample relationships in different gut sections. Feed microbiota was dominated by *Proteobacteria*. This explains the prevalence of *Proteobacteria* in gizzard. *Actinobacteriota*, *Firmicutes* and *Bacteroidota* were dominant phyla in gizzard, jejunum, and caecum. The predominant phyla in our study were similar to previous studies [38,72,73]. The phylum *Firmicutes* consists of mostly beneficial genera, such as *Lactobacillus* and *Bacillus*, and *Bacteroidetes* contains several abundant commensal genera, such as *Bacteroides* and *Prevotella* [74]. These genera play crucial parts in creating a balanced gut microbiota.

DEX produces a significant difference (*p* < 0.05) by both PERMANOVA and paired MANOVA of the caecum. This is expected and confirmed by a separation in the DEX and no DEX groups seen in DAPC plots. DEX provides strong immunosuppression [61,75] and increases broilers’ gut permeability and glucose absorption [42,76]. This impacts gut motility, mast cell activity, mucin production, and, eventually, the composition of gut microbiota [77]. The differences in gizzard were not statistically significant in either PERMANOVA or paired MANOVA.

LEfSe identified genera of commensal and probiotic bacteria differential between groups in the caecum (*p* < 0.05). Birds supplemented with probiotics had higher *Bacteroidetes* abundance. This agrees with Wang et al., [78], who identified a correlation of *Bacillus* sp. and *Bacteroidetes* colonisation in the caecum, producing more propionate, butyrate and isobutyrate content in broilers. This SCFA production in broiler GIT significantly contributes to host energy metabolism and maintaining a healthy gut. This means that caecal microbiota can degrade cellulose and indigestible polysaccharides, producing SCFAs as metabolites [79]. A higher amount of SCFAs means more hydrogen ions (H^+^) produced and a lower hindgut pH, creating an acidic environment. This prevents colonisation and the growth of pathogens [80]. The exact mechanism by which DEX promoted *Lactobacillus* and *Bacillus* in our study remains inconclusive. Furthermore, DEX challenge in the present study creates a gut barrier dysfunction [42] and shifts gut microbiota in broilers significantly [81].

Most studies on the substitutes of AGPs, such as probiotics, prebiotics and phytogens, have centred on caecal microbial profiling by sequencing 16S rRNA genes [29,78]. We expanded our analysis to include microbial profiling in gizzard, jejunal content and jejunal mucosal swabs, but we did not detect significant alterations in these gut sections. One plausible explanation may be attributed to the challenging conditions prevailing in the upper GIT, such as gastric acid, bile salts and some degrading enzymes capable of disrupting the mucosal barrier in the broiler GIT [82]. In a similar study, a dose of DEX at 0.35 ppm was administered on days 17, 19 and 23 of age to minimise the severe reduction in broiler performance observed [83]. This dosage was sufficient to induce gut barrier dysfunction and impair broiler growth [81]. The DEX challenge dose used in this study was optimised previously [37] to provide a high, yet safe level of challenge. The challenge induced a chronic health condition, suppressing the entire broiler physiology and GIT systems, overriding the positive effects of probiotics [26].

Several studies have described probiotic benefits in modulating microbial profiles in broilers [29]. Balancing gut microbiota has been proposed as a possible mode of action for probiotics [84]. Therefore, studies of microbial community composition are as important as understanding the impacts of probiotics on production performance for those seeking to understand the mode of action of probiotics.

## 5. Conclusions

Our data suggest that *Bacillus* probiotic supplementation significantly improved the production performance of broilers before DEX challenge. Furthermore, the structure of the gut microbiota underwent alterations in response to the leaky gut challenge, with specific gut sections responding differently. Significant differences were observed in the caecum. Our data also indicate a strong influence of feed microbiota on the gizzard microbial community detectible using 16S amplicon sequencing methodology. Regardless of whether the feed microbiota originates from DNA detected from live or dead bacteria, it appears to introduce background noise that may overshadow the specific organ response to major challenges such as DEX. It would be interesting to observe changes in either the acute DEX challenge or the post-DEX recovery phase to determine whether meaningful outcomes could be achieved from *Bacillus* probiotic supplementation in poultry. This is crucial for identifying the most promising solution to AGPs.

## Figures and Tables

**Table 1:** Broiler performance parameters before the DEX period (days 0–28).

	Experimental Groups		Statistics	
Component	C	P	SEM	*p*-Value
BW 0 (g)	42.38	42.97	0.36	0.11
BW 7 (g)	152.30	155.91	2.26	0.14
BW 14 (g)	416.54	420.24	5.98	0.53
BW 21 (g)	885.46	910.57	11.85	<0.05 *
BW 28 (g)	1573.27	1606.12	19.63	0.12
ADG 0–7 (g)	15.70	16.14	0.34	0.23
ADG7–14 (g)	37.75	37.76	0.94	0.99
ADG 14–21 (g)	66.99	70.05	1.02	<0.05 *
ADG 21–28 (g)	98.26	99.36	1.80	0.57
ADG 0–28 (g)	56.19	57.36	0.70	0.12
ADFI 0–7 (g)	16.47	16.52	0.35	0.90
ADFI 7–14 (g)	46.50	46.78	0.83	0.74
ADFI 14–21 (g)	83.49	87.58	1.09	<0.05 *
ADFI 21–28 (g)	138.12	138.69	2.18	0.80
ADFI 0–28 (g)	71.95	72.47	0.98	0.63
FCR 0–7	0.76	0.74	0.01	0.17
FCR 7–14	1.23	1.24	0.02	0.58
FCR 14–21	1.25	1.25	0.01	0.70
FCR 21–28	1.41	1.40	0.01	0.29
FCR 0–28	1.28	1.26	0.00	<0.05 *
Mortality (%)	3.13	1.56		

* Statistically significant *p* < 0.05.

**Table 2:** Broiler performance post-DEX period (days 28–35).

	Experimental Groups				
Component	C	P	CD	PD	*p*-Value
BW 35 (g)	2275.61 ^a^	2296.75 ^a^	1836.80 ^b^	1883.95 ^b^	<0.05
BWG 28–35 (g)	98.39 ^a^	101.02 ^a^	39.60 ^b^	37.33 ^b^	<0.05
FCR 28–35 (g)	1.68 ^a^	1.65 ^a^	4.40 ^b^	4.71 ^b^	<0.05
Mortality (%)	0.00	0.00	1.00	1.00	

^a,b^ Mean values within a row with unlike superscript letters were significantly different (*p* ≤ 0.05).

**Table 3:** PERMANOVA of caecum by both weighted and unweighted Unifrac.

Groups	R^2^	*p*-Value	Distance
DEX	0.097489	0.001 *	Weighted Unifrac
Probiotic	0.017196	0.115	Weighted Unifrac
Treatment	0.01278	0.267	Weighted Unifrac
Probiotic	0.020812	0.013 *	Unweighted Unifrac
DEX	0.047365	0.001 *	Unweighted Unifrac
Treatment	0.01497	0.125	Unweighted Unifrac

DEX ×Probiotic interactions were not significant (*p* > 0.9) by either weighted or unweighed Unifrac. Treatment PERMANOVA compares the difference between the four groups (C, CD, P and PD). * Statistically significant (*p* < 0.05).

**Table 4:** Paired MANOVA of relevant experimental group comparisons in the caecum. There was a significant difference between groups in both unweighted and weighted Unifrac.

Groups	R2	Pr (>F)	Distance
C vs. CD	0.081011	0.001 *	Unweighted Unifrac
C vs. P	0.025188	0.318	Unweighted Unifrac
CD vs. PD	0.052213	0.007 *	Unweighted Unifrac
P vs. PD	0.047553	0.005 *	Unweighted Unifrac
C vs. CD	0.161935	0.001 *	Weighted Unifrac
C vs. P	0.014989	0.685	Weighted Unifrac
CD vs. PD	0.056141	0.015 *	Weighted Unifrac
P vs. PD	0.063808	0.008 *	Weighted Unifrac

* Statistically significant *p* < 0.05.

## Data Availability

The raw sequence data are available from the NCBI SRA database with accession number PRJNA1068165 (https://www.ncbi.nlm.nih.gov/bioproject/1068165, accessed on 23 January 2024).
